# Supplementary material for: The Relationships Between Healthcare Access, Gender, and Psychedelics and Their Effects on Distress
Source: Healthcare (Basel). 2025 May 16;13(10):1158. doi: 10.3390/healthcare13101158 (PMC12110895; doi:10.3390/healthcare13101158)
Supplement: Supplementary file 1 [file healthcare-13-01158-s001.zip › healthcare-3566888-supplementary.pdf]

Supplemental Table 1. Mean of Dependent, Independent, and Control Variables by Gender, Private Health Insurance, and Public Health Insurance (Weighted)

	Men	Women	No Private Insurance	Private Insurance	No Public Insurance	Public Insurance
Psychological Distress in Last Month (K6)	9.37	9.72	10.99	8.87	9.50	9.81
	(9.30-9.44)	(9.65-9.79)	(10.90-11.08)	(8.81-8.92)	(9.44 - 9.55)	(9.70 - 9.90)
Women			0.52	0.52	0.50	0.56
			(0.52-0.52)	(0.51-0.52)	(0.49 - 0.50)	(0.55 - 0.56)
Health Insurance						
Uninsured	0.15	0.11	0.39	0.00	0.19	0.00
	(0.15-0.15)	(0.11-0.12)	(0.39-0.39)	(0.00 - 0.00)	(0.19 - 0.20)	(0.00 - 0.00)
Private	0.67	0.66			0.78	0.43
	(0.66-0.67)	(0.66-0.67)		(0.77 - 0.78)	(0.42 - 0.44)	
Public	0.30	0.35	0.55	0.21		
	(0.29-0.30)	(0.35-0.36)	(0.55-0.55)	(0.21-0.21)		
Other	0.02	0.02	0.06	0.00	0.03	0.00
	(0.02-0.02)	(0.02-0.02)	(0.06-0.06)	(0.00-0.00)	(0.03 - 0.03)	(0.00 - 0.00)
Lifetime Psychedelic Use						
MDMA	0.08	0.06	0.08	0.06	0.09	0.04
	(0.08-0.09)	(0.06-0.06)	(0.08-0.09)	(0.06-0.07)	(0.08 - 0.09)	(0.04 - 0.04)
Psilocybin	0.13	0.06	0.09	0.09	0.11	0.06
	(0.13-0.13)	(0.06-0.06)	(0.09-0.10)	(0.09-0.09)	(0.11 - 0.11)	(0.05 - 0.06)
DMT	0.00	0.00	0.00	0.00	0.00	0.00
	(0.00-0.00)	(0.00-0.00)	(0.00-0.00)	(0.00-0.00)	(0.00 - 0.00)	(0.00 - 0.00)
Ayahuasca	0.00	0.00	0.00	0.00	0.00	0.00
	(0.00-0.00)	(0.00-0.00)	(0.00-0.00)	(0.00-0.00)	(0.00 - 0.00)	(0.00 - 0.00)
Peyote/Mescaline	0.06	0.03	0.05	0.04	0.05	0.04
	(0.06-0.06)	(0.03-0.03)	(0.04-0.05)	(0.04-0.04)	(0.04 - 0.05)	(0.03 - 0.04)
LSD	0.14	0.07	0.11	0.10	0.12	0.08
	(0.13-0.14)	(0.07-0.08)	(0.11-0.11)	(0.10-0.10)	(0.11 - 0.12)	(0.07 - 0.08)
Classic Psychedelics	0.18	0.10	0.14	0.14	0.16	0.09
	(0.18-0.18)	(0.10-0.10)	(0.14-0.14)	(0.14-0.14)	(0.15 - 0.16)	(0.09 - 0.10)
Age	8.60	8.74	8.39	8.81	8.20	9.64
	(8.58-8.61)	(8.73-8.75)	(8.37-8.40)	(8.80-8.83)	(8.19 - 8.22)	(9.62 - 9.65)
Educational Attainment	2.73	2.77	2.27	3.00	2.88	2.49
	(2.72-2.73)	(2.77-2.78)	(2.26-2.28)	(2.99-3.00)	(2.87 - 2.89)	(2.47 - 2.50)

Family Income	5.12	4.82	3.64	5.63	5.35	4.16
	(5.10-5.14)	(4.80-4.84)	(3.62-3.66)	(5.62-5.65)	(5.33 - 5.37)	(4.13 - 4.18)
Marital Status						
Single, never Married	0.30	0.25	0.37	0.23	0.31	0.20
	(0.30-0.31)	(0.25-0.25)	(0.37-0.38)	(0.23-0.23)	(0.63 - 0.64)	(0.31 - 0.33)
Married	0.55	0.51	0.36	0.61	0.55	0.48
	(0.54-0.55)	(0.50-0.51)	(0.36-0.37)	(0.60-0.61)	(0.31 - 0.32)	(0.20 - 0.21)
Widowed	0.03	0.09	0.07	0.05	0.02	0.15
	(0.03-0.03)	(0.09-0.09)	(0.07-0.08)	(0.05-0.05)	(0.54 - 0.55)	(0.47 - 0.49)
Divorced/Separated	0.12	0.16	0.19	0.11	0.12	0.17
	(0.12-0.12)	(0.16-0.16)	(0.19-0.19)	(0.11-0.11)	(0.01 - 0.02)	(0.14 - 0.15)
Children	0.49	0.58	0.54	0.53	0.64	0.32
	(0.49-0.49)	(0.57-0.58)	(0.53-0.55)	(0.52-0.54)	(0.12 - 0.13)	(0.16 - 0.17)
Race						
White	0.66	0.65	0.52	0.73	0.65	0.67
	(0.65-0.66)	(0.65-0.66)	(0.51-0.52)	(0.72-0.73)	(0.64 - 0.65)	(0.66 - 0.67)
Black	0.11	0.12	0.17	0.09	0.11	0.14
	(0.11-0.11)	(0.12-0.12)	(0.17-0.17)	(0.09-0.09)	(0.10 - 0.11)	(0.13 - 0.14)
Hispanic	0.16	0.15	0.24	0.10	0.16	0.13
	(0.15-0.16)	(0.14-0.15)	(0.24-0.25)	(0.10-0.10)	(0.16 - 0.16)	(0.12 - 0.13)
Asian	0.05	0.05	0.04	0.06	0.06	0.03
	(0.05-0.05)	(0.05-0.05)	(0.04-0.04)	(0.06-0.06)	(0.05 - 0.06)	(0.03 - 0.03)
Hawaiian/Pacific Islander	0.00	0.00	0.00	0.00	0.00	0.01
	(0.00-0.00)	(0.00-0.00)	(0.004-0.004)	(0.003-0.003)	(0.00 - 0.00)	(0.00 - 0.00)
Native American	0.00	0.01	0.01	0.00	0.01	0.02
	(0.00-0.01)	(0.00-0.01)	(0.01-0.01)	(0.003-0.003)	(0.00 - 0.00)	(0.00 - 0.01)
Two or More Race (non-Hispanic)	0.01	0.02	0.02	0.01	4.75	5.29
	(0.01-0.01)	(0.01-0.02)	(0.02-0.02)	(0.01-0.01)	(0.01 - 0.01)	(0.01 - 0.02)
Religiosity	4.63	5.20	5.01	4.88	1.84	2.00
	(4.61-4.65)	(5.18-5.21)	(5.00-5.03)	(4.86-4.90)	(4.73 - 4.76)	(5.26 - 5.31)
Religious Attendance in Days	1.69	2.09	1.65	2.02	0.18	0.12
	(1.67-1.70)	(2.07-2.10)	(1.63-1.66)	(2.00-2.03)	(1.83 - 1.85)	(1.98 - 2.01)
Lifetime Drug Use						
Cocaine	0.20	0.12	0.18	0.15	0.52	0.35
	(0.20-0.21)	(0.12-0.13)	(0.17-0.18)	(0.15-0.15)	(0.18 - 0.19)	(0.11 - 0.12)
Marijuana	0.52	0.42	0.45	0.47	0.03	0.03

	(0.51-0.52)	(0.41-0.42)	(0.44-0.45)	(0.47-0.47)	(0.51 - 0.52)	(0.34 - 0.35)
PCP	0.04	0.02	0.03	0.02	0.10	0.06
	(0.04-0.04)	(0.02-0.02)	(0.03-0.03)	(0.02-0.02)	(0.02 - 0.03)	(0.02 - 0.03)
Inhalants	0.12	0.06	0.09	0.09	0.11	0.08
	(0.12-0.12)	(0.06-0.06)	(0.08-0.09)	(0.08-0.09)	(0.10 - 0.11)	(0.05 - 0.06)
Stimulants	0.11	0.09	0.10	0.10	0.08	0.10
	(0.11-0.11)	(0.09-0.10)	(0.09-0.10)	(0.10-0.10)	(0.11 - 0.12)	(0.07 - 0.08)
Sedatives	0.08	0.09	0.08	0.08	0.16	0.17
	(0.07-0.08)	(0.08-0.09)	(0.08-0.08)	(0.08-0.08)	(0.07 - 0.08)	(0.09 - 0.10)
Tranquilizer	0.15	0.18	0.17	0.16	0.02	0.02
	(0.14-0.15)	(0.17-0.18)	(0.16-0.17)	(0.16-0.16)	(0.16 - 0.17)	(0.17 - 0.18)
Heroine	0.03	0.01	0.03	0.01	0.36	0.36
	(0.03-0.03)	(0.01-0.01)	(0.03-0.03)	(0.01-0.01)	(0.01 - 0.02)	(0.02 - 0.02)
Pain Relievers	0.36	0.36	0.35	0.37	0.57	0.57
	(0.36-0.37)	(0.35-0.36)	(0.34-0.35)	(0.36-0.37)	(0.36 - 0.36)	(0.35 - 0.36)
Tobacco	0.71	0.44	0.57	0.57	2.76	3.12
	(0.71-0.72)	(0.44-0.45)	(0.57-0.58)	(0.57-0.58)	(0.57 - 0.58)	(0.56 - 0.57)
Age of First Alcohol Use	2.66	3.07	3.02	2.81	1.70	1.48
	(2.65-2.67)	(3.07-3.08)	(3.00-3.03)	(2.80-2.81)	(2.75 - 2.77)	(3.10 - 3.12)
Thrill Seeking Behavior	1.80	1.47	1.57	1.66	1.70	1.48
	(1.79-1.80)	(1.47-1.48)	(1.56-1.58)	(1.65-1.67)	(1.70 - 1.71)	(1.47 - 1.48)

Source: 2008-2019 National Survey of Drug Use and Health, n=*484,732*

[a]. Calculated with T-Tests

[b]. Standard deviations in parentheses.

$^{\dagger}p < 0.10$, $^{*}p < 0.05$, $^{**}p < 0.01$, $^{***}p < 0.001$ (two-tailed)

Supplemental Table 2. Mean Difference of Dependent, Independent, and Control Variables by Gender (Weighted)

	Men (-) Women		No Private Health Insurance (-) Private Health Insurance		No Public Health Insurance (-) Public Health Insurance	
Psychological Distress in Last Month (K6)	-0.36	***	2.12	***	-0.31	***
	(-0.45--0.26)		(2.02-2.22)		(-0.43 - -0.19)	
Women			0.01	**	-0.06	***
			(0.00-0.01)		(-0.07 - -0.06)	
Health Insurance						
Uninsured	0.03	***	0.39	***	0.19	***
	(0.03-0.04)		(0.39-0.39)		(0.19 - 0.20)	
Private	0.01	**			0.35	***
	(0.00-0.01)				(0.34 - 0.35)	
Public	-0.06	***	0.34	***		
	(-0.06--0.05)		(0.34-0.34)			
Other	0.00		0.06	***	0.03	***
	(-0.00-0.00)		(0.06-0.06)		(0.03 - 0.03)	
Lifetime Psychedelic Use						
MDMA	0.03	***	0.02	***	0.04	***
	(0.03-0.03)		(0.02-0.02)		(0.04 - 0.05)	
Psilocybin	0.07	***	0.00		0.05	***
	(0.07-0.07)		(-0.00-0.00)		(0.05 - 0.06)	
DMT	0.00	***	0.00	*	0.00	***
	(0.00-0.00)		(0.00-0.00)		(0.00 - 0.00)	
Ayahuasca	0.00		0.00		0.00	*
	(-0.00-0.00)		(-0.00-0.00)		(0.00 - 0.00)	
Peyote/Mescaline	0.03	***	0.00	***	0.01	***
	(0.03-0.04)		(0.00-0.01)		(0.00 - 0.01)	
LSD	0.06	***	0.01	***	0.04	***
	(0.06-0.07)		(0.01-0.01)		(0.04 - 0.05)	
Classic Psychedelics	0.08	***	0.01	**	0.06	***
	(0.08-0.08)		(0.00-0.01)		(0.06 - 0.07)	
Age	-0.14	***	-0.43	***	-1.43	***
	(-0.16--0.13)		(-0.45--0.41)		(-1.45 - -1.41)	
Educational Level	-0.05	***	-0.73	***	0.39	***
	(-0.06--0.04)		(-0.74--0.72)		(0.38 - 0.40)	

Family Income	0.30 ***	-1.99 ***	1.19 ***
	(0.28-0.32)	(-2.01--1.97)	(1.17 - 1.22)
Marital Status			
Single, never Married	0.06 ***	0.14 ***	0.32 ***
	(0.05-0.06)	(0.14-0.15)	(0.31 - 0.33)
Married	0.04 ***	-0.24 ***	0.11 ***
	(0.04-0.04)	(-0.25--0.24)	(0.11 - 0.11)
Widowed	-0.06 ***	0.02 ***	0.07 ***
	(-0.06--0.06)	(0.02-0.02)	(0.06 - 0.07)
Divorced/Separated	-0.04 ***	0.08 ***	-0.13 ***
	(-0.04--0.03)	(0.08-0.08)	(-0.13 - -0.13)
Children	-0.09 ***	0.01 **	-0.05 ***
	(-0.09--0.08)	(0.00-0.02)	(-0.05 - -0.04)
Race			
White	0.01 **	-0.21 ***	-0.02 ***
	(0.01-0.01)	(-0.22--0.21)	(-0.02 - -0.01)
Black	-0.01 ***	0.08 ***	-0.03 ***
	(-0.02--0.01)	(0.08-0.08)	(-0.03 - -0.03)
Hispanic	0.01 ***	0.14 ***	0.03 ***
	(0.01-0.02)	(0.13-0.14)	(0.03 - 0.04)
Asian	0.00 *	-0.02 ***	0.03 ***
	(-0.01--0.00)	(-0.02--0.02)	(0.03 - 0.03)
Hawaiian/Pacific Islander	0.00	0.00 ***	0.00
	(-0.00-0.00)	(0.00-0.00)	(0.00 - 0.00)
Native American	0.00 †	0.01 ***	0.00 ***
	(-0.01--0.00)	(0.01-0.01)	(-0.00 - -0.00)
Two or More Race (non-Hispanic)	0.00	0.01 ***	-0.01 ***
	(-0.01--0.00)	(0.01-0.01)	(-0.01 - -0.00)
Religiosity	-0.57 ***	0.13 ***	-0.54 ***
	(-0.59--0.55)	(0.10-0.15)	(-0.57 - -0.52)
Religious Attendance	-0.40 ***	-0.37 ***	-0.16 ***
	(-0.41--0.38)	(-0.39--0.35)	(-0.18 - -0.14)
Lifetime Drug Use			
Cocaine	0.08 ***	0.03 ***	0.07 ***
	(0.07-0.08)	(0.02-0.03)	(0.06 - 0.07)
Marijuana	0.10 ***	-0.02 ***	0.17 ***

	(0.10-0.11)	(-0.03--0.02)	(0.17 - 0.18)
PCP	0.02 ***	0.01 ***	0.00 **
	(0.02-0.02)	(0.01-0.01)	(0.00 - 0.00)
Inhalants	0.06 ***	0.00	0.05 ***
	(0.06-0.06)	(-0.00-0.00)	(0.04 - 0.05)
Stimulants	0.01 ***	-0.01 **	0.04 ***
	(0.01-0.02)	(-0.01--0.00)	(0.04 - 0.04)
Sedatives	-0.01 ***	0.00 ***	-0.02 ***
	(-0.01--0.01)	(-0.01--0.00)	(-0.02 - -0.02)
Tranquilizer	-0.03 ***	0.00 *	-0.01 ***
	(-0.03--0.03)	(0.00-0.01)	(-0.02 - -0.01)
Heroine	0.02 ***	0.02 ***	0.00 ***
	(0.02-0.02)	(0.02-0.02)	(-0.01 - -0.00)
Pain Relievers	0.01 **	-0.02 ***	0.00
	(0.01-0.01)	(-0.03--0.02)	(0.00 - 0.00)
Tobacco	0.27 ***	0.00	0.00 *
	(0.27-0.28)	(-0.01-0.00)	(-0.00 - 0.01)
Age of First Alcohol Use	-0.41 ***	0.21 ***	-0.35 ***
	(-0.42--0.40)	(0.20-0.22)	(-0.37 - -0.34)
Thrill Seeking Behavior	0.33 ***	-0.09 ***	0.22 ***
	(0.32-0.33)	(-0.10--0.08)	(0.22 - 0.23)

Source: 2008-2019 National Survey of Drug Use and Health, n=*484,732*

[a]. Calculated with T-Tests

[b]. Standard deviations in parentheses.

†$p < 0.10$, *$p < 0.05$, **$p < 0.01$, ***$p < 0.001$ (two-tailed)

Supplemental Table 3. Weighted Multivariate Ordinary Least Square Regression Predicting Psychological Distress Among the Total Population and By Men and Women

	Total Population				Men				Women			
	Model 1	Model 2	Model 3	Model 4	Model 5	Model 6	Model 7	Model 8	Model 9	Model 10	Model 11	Model 12
Age	-0.455***	-0.468***	-0.453***	-0.466***	-0.343***	-0.376***	-0.375***	-0.374***	-0.514***	-0.514***	-0.529***	-0.513***
	(0.00961)	(0.00960)	(0.00977)	(0.00977)	(0.0182)	(0.0186)	(0.0185)	(0.0189)	(0.0131)	(0.0133)	(0.0134)	(0.0133)
Family Income	-0.371***	-0.288***	-0.372***	-0.289***	-0.370***	-0.287***	-0.338***	-0.288***	-0.364***	-0.286***	-0.345***	-0.287***
	(0.0124)	(0.0129)	(0.0124)	(0.0129)	(0.0212)	(0.0207)	(0.0207)	(0.0207)	(0.0155)	(0.0169)	(0.0161)	(0.0169)
Education Level	-0.386***	-0.299***	-0.383***	-0.295***	-0.389***	-0.300***	-0.352***	-0.301***	-0.411***	-0.328***	-0.385***	-0.328***
	(0.0240)	(0.0243)	(0.0243)	(0.0246)	(0.0404)	(0.0427)	(0.0416)	(0.0429)	(0.0289)	(0.0297)	(0.0300)	(0.0301)
[a]Women	0.963***	0.974***	0.953***	0.965***								
	(0.0462)	(0.0461)	(0.0458)	(0.0457)								
[b]Married	-0.665***	-0.581***	-0.664***	-0.580***	-0.899***	-0.836***	-0.927***	-0.831***	-0.487***	-0.417***	-0.471***	-0.416***
	(0.0654)	(0.0644)	(0.0655)	(0.0645)	(0.103)	(0.105)	(0.104)	(0.105)	(0.0763)	(0.0759)	(0.0765)	(0.0759)
Widowed	-0.635***	-0.586***	-0.637***	-0.587***	-0.202	-0.241	-0.398	-0.237	-0.540***	-0.468**	-0.607***	-0.481**
	(0.119)	(0.120)	(0.120)	(0.120)	(0.256)	(0.261)	(0.263)	(0.260)	(0.148)	(0.149)	(0.150)	(0.149)
Divorced	0.290**	0.288**	0.291**	0.289**	0.143	0.140	0.127	0.149	0.424***	0.408***	0.429***	0.415***
	(0.0921)	(0.0908)	(0.0926)	(0.0913)	(0.149)	(0.148)	(0.149)	(0.148)	(0.108)	(0.107)	(0.109)	(0.108)
Children	0.271***	0.245***	0.267***	0.241***	0.173***	0.192***	0.219***	0.191***	0.320***	0.277***	0.323***	0.276***
	(0.0240)	(0.0248)	(0.0240)	(0.0248)	(0.0448)	(0.0464)	(0.0455)	(0.0459)	(0.0266)	(0.0272)	(0.0266)	(0.0269)
[c]Black	-0.464***	-0.550***	-0.468***	-0.554***	-0.289**	-0.368***	-0.302**	-0.362***	-0.537***	-0.627***	-0.544***	-0.629***
	(0.0706)	(0.0694)	(0.0702)	(0.0691)	(0.103)	(0.105)	(0.104)	(0.105)	(0.0999)	(0.0986)	(0.0993)	(0.0984)
Hispanic	-0.0366	-0.156*	-0.0377	-0.157*	0.0211	-0.0605	0.0447	-0.0548	-0.0570	-0.198*	-0.0487	-0.191*
	(0.0767)	(0.0767)	(0.0768)	(0.0769)	(0.120)	(0.118)	(0.119)	(0.117)	(0.0940)	(0.0955)	(0.0949)	(0.0959)
Asian	0.336**	0.317**	0.335**	0.316**	0.437*	0.436*	0.474**	0.437*	0.242†	0.212	0.263†	0.210
	(0.101)	(0.0993)	(0.101)	(0.0996)	(0.170)	(0.168)	(0.171)	(0.168)	(0.134)	(0.131)	(0.135)	(0.132)
[d]NI/AN	0.749**	0.650*	0.782**	0.686**	1.210*	1.120*	1.156*	1.086*	0.474	0.373	0.450	0.325
	(0.258)	(0.257)	(0.259)	(0.259)	(0.470)	(0.466)	(0.473)	(0.468)	(0.352)	(0.363)	(0.350)	(0.360)
[e]NHOPI	0.372	0.269	0.371	0.267	0.308	0.202	0.352	0.228	0.346	0.268	0.349	0.271
	(0.416)	(0.416)	(0.416)	(0.416)	(0.679)	(0.699)	(0.690)	(0.695)	(0.456)	(0.454)	(0.452)	(0.454)
Multi-Race/Other	0.330*	0.255†	0.341*	0.266†	0.349†	0.276	0.297	0.279	0.306	0.232	0.279	0.232
	(0.152)	(0.151)	(0.151)	(0.150)	(0.206)	(0.207)	(0.205)	(0.207)	(0.210)	(0.205)	(0.209)	(0.205)
Religious Salience	-0.0448***	-0.0499***	-0.0452***	-0.0504***	-0.0527**	-0.0586***	-0.0541**	-0.0587***	-0.0422**	-0.0464**	-0.0430**	-0.0466**
	(0.0111)	(0.0110)	(0.0111)	(0.0110)	(0.0164)	(0.0164)	(0.0163)	(0.0163)	(0.0151)	(0.0150)	(0.0150)	(0.0150)
Religious	-0.130***	-0.119***	-0.130***	-0.119***	-0.0768***	-0.0686**	-0.0774***	-0.0692**	-0.149***	-0.138***	-0.149***	-0.139***

Attendance												
	(0.0147)	(0.0147)	(0.0147)	(0.0147)	(0.0220)	(0.0219)	(0.0220)	(0.0219)	(0.0195)	(0.0196)	(0.0196)	(0.0197)
Cocaine	-0.0829	-0.106	-0.0619	-0.0845	-0.208*	-0.213*	-0.207†	-0.215*	-0.00718	-0.0405	0.0150	-0.0111
	(0.0678)	(0.0675)	(0.0691)	(0.0687)	(0.104)	(0.104)	(0.107)	(0.107)	(0.0865)	(0.0865)	(0.0872)	(0.0871)
Stimulant	0.627***	0.644***	0.635***	0.652***	0.657***	0.668***	0.655***	0.668***	0.635***	0.654***	0.645***	0.662***
	(0.0685)	(0.0682)	(0.0685)	(0.0682)	(0.106)	(0.106)	(0.106)	(0.106)	(0.0855)	(0.0852)	(0.0859)	(0.0856)
Sedative	0.869***	0.853***	0.888***	0.873***	0.543***	0.512***	0.514***	0.546***	1.060***	1.055***	1.053***	1.064***
	(0.0724)	(0.0722)	(0.0727)	(0.0725)	(0.113)	(0.111)	(0.112)	(0.112)	(0.0993)	(0.0998)	(0.0990)	(0.0996)
Tranquilizer	1.050***	1.027***	1.049***	1.026***	0.968***	0.942***	0.940***	0.934***	1.117***	1.094***	1.109***	1.096***
	(0.0545)	(0.0552)	(0.0546)	(0.0552)	(0.0892)	(0.0909)	(0.0886)	(0.0896)	(0.0796)	(0.0805)	(0.0800)	(0.0802)
Heroine	0.696***	0.594***	0.727***	0.627***	0.905***	0.806***	0.838***	0.821***	0.537*	0.438†	0.517*	0.473*
	(0.149)	(0.147)	(0.147)	(0.145)	(0.188)	(0.187)	(0.186)	(0.186)	(0.231)	(0.227)	(0.231)	(0.225)
Pain Killers	0.353***	0.339***	0.355***	0.341***	0.321***	0.317***	0.328***	0.317***	0.406***	0.386***	0.410***	0.392***
	(0.0606)	(0.0611)	(0.0607)	(0.0611)	(0.0876)	(0.0871)	(0.0884)	(0.0876)	(0.0755)	(0.0765)	(0.0753)	(0.0759)
Marijuana	0.406***	0.413***	0.401***	0.408***	0.382***	0.416***	0.420***	0.412***	0.412***	0.404***	0.424***	0.404***
	(0.0684)	(0.0677)	(0.0686)	(0.0679)	(0.107)	(0.106)	(0.105)	(0.105)	(0.0783)	(0.0777)	(0.0785)	(0.0779)
PCP	-0.0149	-0.0474	0.0785	0.0505	-0.440†	-0.463*	-0.483*	-0.379†	0.480*	0.446*	0.488*	0.529*
	(0.183)	(0.181)	(0.182)	(0.179)	(0.227)	(0.224)	(0.224)	(0.225)	(0.226)	(0.221)	(0.224)	(0.228)
Inhalants	0.512***	0.508***	0.536***	0.531***	0.625***	0.627***	0.633***	0.631***	0.470***	0.465***	0.495***	0.494***
	(0.0733)	(0.0729)	(0.0732)	(0.0728)	(0.106)	(0.106)	(0.107)	(0.107)	(0.104)	(0.105)	(0.104)	(0.105)
Tobacco	0.434***	0.406***	0.431***	0.403***	0.00775	-0.00719	-0.0172	-0.0126	0.581***	0.548***	0.570***	0.548***
	(0.0547)	(0.0547)	(0.0546)	(0.0547)	(0.0936)	(0.0932)	(0.0935)	(0.0927)	(0.0643)	(0.0642)	(0.0641)	(0.0643)
Age of 1st Alcohol Use	-0.0273	-0.0355	-0.0272	-0.0354	0.0142	0.0109	0.00966	0.0116	-0.0262	-0.0372	-0.0340	-0.0378
	(0.0270)	(0.0269)	(0.0270)	(0.0269)	(0.0413)	(0.0409)	(0.0415)	(0.0408)	(0.0348)	(0.0346)	(0.0346)	(0.0347)
Thrill Seeking	0.764***	0.773***	0.766***	0.775***	0.635***	0.648***	0.643***	0.648***	0.903***	0.908***	0.904***	0.911***
	(0.0332)	(0.0332)	(0.0331)	(0.0331)	(0.0513)	(0.0515)	(0.0515)	(0.0514)	(0.0442)	(0.0436)	(0.0440)	(0.0435)
[f]2009	-0.0411	-0.0561	-0.0402	-0.0552	0.197	0.175	0.199	0.184	-0.178	-0.184	-0.185	-0.188
	(0.118)	(0.119)	(0.119)	(0.119)	(0.219)	(0.220)	(0.221)	(0.222)	(0.160)	(0.160)	(0.161)	(0.160)
2010	0.0587	0.0332	0.0595	0.0341	0.157	0.121	0.147	0.120	-0.00111	-0.0165	-0.00293	-0.0182
	(0.116)	(0.116)	(0.116)	(0.116)	(0.152)	(0.155)	(0.151)	(0.155)	(0.155)	(0.154)	(0.154)	(0.154)
2011	0.0648	0.0294	0.0669	0.0315	0.305†	0.250	0.290†	0.250	-0.0671	-0.0868	-0.0724	-0.0859
	(0.115)	(0.116)	(0.115)	(0.116)	(0.173)	(0.174)	(0.173)	(0.176)	(0.151)	(0.152)	(0.151)	(0.152)
2012	0.164	0.134	0.165	0.136	0.450**	0.403*	0.428*	0.399*	-0.00983	-0.0255	-0.0161	-0.0262

	(0.115)	(0.115)	(0.116)	(0.116)	(0.169)	(0.168)	(0.168)	(0.169)	(0.151)	(0.152)	(0.150)	(0.152)
2013	0.293*	0.265†	0.294*	0.266†	0.533**	0.488*	0.505*	0.487*	0.154	0.142	0.136	0.139
	(0.138)	(0.138)	(0.137)	(0.138)	(0.199)	(0.200)	(0.198)	(0.200)	(0.181)	(0.181)	(0.180)	(0.181)
2014	0.122	0.0863	0.118	0.0823	0.246	0.183	0.189	0.172	0.0534	0.0373	0.0308	0.0260
	(0.102)	(0.104)	(0.103)	(0.104)	(0.180)	(0.183)	(0.182)	(0.183)	(0.141)	(0.142)	(0.141)	(0.142)
2015	-0.279*	-0.302*	-0.281*	-0.304*	-0.0251	-0.0772	-0.0790	-0.0815	-0.483**	-0.486**	-0.517**	-0.502**
	(0.116)	(0.117)	(0.116)	(0.117)	(0.170)	(0.170)	(0.170)	(0.170)	(0.164)	(0.166)	(0.165)	(0.165)
2016	-0.161	-0.179	-0.165	-0.184†	0.241	0.186	0.179	0.181	-0.471**	-0.460**	-0.509**	-0.476**
	(0.110)	(0.110)	(0.110)	(0.110)	(0.162)	(0.162)	(0.162)	(0.162)	(0.152)	(0.154)	(0.154)	(0.154)
2017	-0.00409	-0.0349	-0.0106	-0.0417	0.426*	0.372*	0.363*	0.363*	-0.306†	-0.318†	-0.347*	-0.332*
	(0.117)	(0.118)	(0.116)	(0.118)	(0.169)	(0.171)	(0.169)	(0.171)	(0.161)	(0.162)	(0.161)	(0.161)
2018	0.283*	0.250*	0.278*	0.245*	0.797***	0.734***	0.731***	0.726***	-0.0904	-0.102	-0.131	-0.118
	(0.121)	(0.122)	(0.120)	(0.121)	(0.180)	(0.183)	(0.181)	(0.183)	(0.161)	(0.162)	(0.162)	(0.161)
2019	0.601***	0.554***	0.593***	0.546***	1.055***	0.988***	0.984***	0.975***	0.262†	0.231	0.219	0.216
	(0.125)	(0.126)	(0.125)	(0.126)	(0.188)	(0.190)	(0.189)	(0.190)	(0.157)	(0.159)	(0.158)	(0.158)
MDMA	0.172*	0.150†	0.170*	0.147†	0.219†	0.205†	0.212†	0.162	0.136	0.115	0.162	0.122
	(0.0847)	(0.0837)	(0.0853)	(0.0843)	(0.116)	(0.115)	(0.115)	(0.119)	(0.113)	(0.113)	(0.114)	(0.113)
LCPU	-0.193**	-0.187**			-0.150	-0.147			-0.156	-0.156		
	(0.0663)	(0.0659)			(0.114)	(0.114)			(0.100)	(0.0994)		
Priv Hlth Ins		-0.977***		-0.979***		-0.815***		-0.767***		-1.056***		-0.987***
		(0.0625)		(0.0624)		(0.0896)		(0.0849)		(0.0765)		(0.0717)
Pub Hlth Ins		0.118*		0.117*		0.399***	0.670***	0.436***		-0.0679	0.332***	-0.0151
		(0.0567)		(0.0569)		(0.0952)	(0.0909)	(0.0947)		(0.0775)	(0.0727)	(0.0785)
Oth Hlth Ins		-0.628***		-0.631***		-0.491*	0.0453			-0.731***	-0.000542	
		(0.141)		(0.141)		(0.215)	(0.203)			(0.181)	(0.165)	
Psilocybin			-0.304***	-0.296***			-0.247†	-0.194			-0.270*	-0.232†
			(0.0843)	(0.0839)			(0.132)	(0.133)			(0.124)	(0.123)
DMT			0.673	0.660			1.183†	1.204†			-0.324	-0.288
			(0.586)	(0.578)			(0.657)	(0.643)			(1.402)	(1.426)
Ayahuasca			1.455	1.397			1.855	2.025			0.515	0.620
			(1.674)	(1.673)			(2.536)	(2.524)			(2.009)	(2.088)
Pey/Mesc			-0.451***	-0.472***				-0.486*				-0.408*
			(0.126)	(0.124)				(0.187)				(0.178)

LSD			0.118	0.125			0.137	0.220†			-0.0252	0.0260
			(0.0833)	(0.0832)			(0.129)	(0.131)			(0.122)	(0.121)
Women * Priv Hlth Ins												
Women * Pub Hlth Ins												
Constant	16.57***	16.79***	16.54***	16.77***	15.36***	15.62***	15.39***	15.54***	18.12***	18.34***	18.10***	18.25***
	(0.207)	(0.205)	(0.209)	(0.207)	(0.365)	(0.369)	(0.366)	(0.375)	(0.233)	(0.229)	(0.232)	(0.230)
Observations	158268	158268	158268	158268	58652	58652	58652	58652	99616	99616	99616	99616
R^2	0.150	0.154	0.150	0.155	0.131	0.136	0.133	0.136	0.165	0.169	0.166	0.169

Standard errors in parentheses
Source: 2008-2019 National Survey of Drug Use and Health, n=484,732
[a]Men serve as the reference category for Women
[b]Single serves as the reference category for Marital Statuses
[c]White serves as the reference category for race/ethnicity
[d]Native Indian or Alaskan Native
[e]Native Hawaiian or Pacific Islander
[f]2008 serves as the reference category for the survey year
† $p < .10$, * $p < .05$, ** $p < .01$, *** $p < .001$

Supplemental Table 4. Weighted Multivariate Ordinary Least Square Regression Predicting Psychological Distress Among the Total Population with Interaction Terms (Psychedelics (x) Private or Public Health Insurance)

	Model 1	Model 2	Model 3	Model 4	Model 5	Model 6	Model 7	Model 8	Model 9	Model 10	Model 11	Model 12	Model 13	Model 14
Age	-0.469***	-0.466***	-0.467***	-0.466***	-0.466***	-0.467***	-0.467***	-0.465***	-0.465***	-0.464***	-0.466***	-0.466***	-0.466***	-0.464***
	(0.00962)	(0.00978)	(0.00979)	(0.00977)	(0.00977)	(0.00980)	(0.00979)	(0.00955)	(0.00975)	(0.00974)	(0.00977)	(0.00977)	(0.00969)	(0.00972)
Family Income	-0.288***	-0.289***	-0.289***	-0.289***	-0.289***	-0.289***	-0.288***	-0.288***	-0.289***	-0.289***	-0.289***	-0.289***	-0.288***	-0.288***
	(0.0129)	(0.0129)	(0.0129)	(0.0129)	(0.0129)	(0.0128)	(0.0129)	(0.0129)	(0.0129)	(0.0128)	(0.0129)	(0.0129)	(0.0129)	(0.0129)
Education Level	-0.299***	-0.295***	-0.295***	-0.295***	-0.295***	-0.295***	-0.296***	-0.305***	-0.297***	-0.299***	-0.295***	-0.295***	-0.297***	-0.301***
	(0.0244)	(0.0246)	(0.0247)	(0.0247)	(0.0246)	(0.0247)	(0.0247)	(0.0243)	(0.0246)	(0.0247)	(0.0246)	(0.0246)	(0.0248)	(0.0247)
[a]Women	0.975***	0.965***	0.965***	0.965***	0.965***	0.965***	0.966***	0.978***	0.962***	0.965***	0.965***	0.965***	0.968***	0.968***
	(0.0462)	(0.0457)	(0.0458)	(0.0457)	(0.0457)	(0.0458)	(0.0458)	(0.0460)	(0.0458)	(0.0456)	(0.0457)	(0.0457)	(0.0458)	(0.0456)
[b]Married	-0.577***	-0.580***	-0.577***	-0.580***	-0.580***	-0.579***	-0.577***	-0.579***	-0.580***	-0.578***	-0.580***	-0.580***	-0.578***	-0.577***
	(0.0649)	(0.0645)	(0.0649)	(0.0645)	(0.0645)	(0.0646)	(0.0649)	(0.0645)	(0.0645)	(0.0646)	(0.0645)	(0.0644)	(0.0643)	(0.0646)
Widowed	-0.579***	-0.587***	-0.584***	-0.587***	-0.587***	-0.586***	-0.581***	-0.548***	-0.574***	-0.563***	-0.587***	-0.587***	-0.577***	-0.560***
	(0.120)	(0.120)	(0.120)	(0.120)	(0.120)	(0.120)	(0.120)	(0.119)	(0.120)	(0.120)	(0.120)	(0.120)	(0.121)	(0.120)
Divorced	0.286**	0.289**	0.289**	0.289**	0.289**	0.289**	0.288**	0.276**	0.289**	0.282**	0.289**	0.289**	0.286**	0.282**
	(0.0909)	(0.0913)	(0.0914)	(0.0913)	(0.0913)	(0.0913)	(0.0913)	(0.0914)	(0.0915)	(0.0916)	(0.0913)	(0.0913)	(0.0914)	(0.0914)
Children	0.246***	0.241***	0.242***	0.241***	0.241***	0.241***	0.242***	0.239***	0.236***	0.237***	0.241***	0.241***	0.240***	0.237***
	(0.0248)	(0.0248)	(0.0248)	(0.0248)	(0.0248)	(0.0248)	(0.0248)	(0.0247)	(0.0247)	(0.0248)	(0.0248)	(0.0248)	(0.0247)	(0.0249)
[c]Black	-0.537***	-0.554***	-0.545***	-0.554***	-0.554***	-0.553***	-0.545***	-0.541***	-0.555***	-0.546***	-0.554***	-0.554***	-0.552***	-0.547***
	(0.0690)	(0.0692)	(0.0690)	(0.0691)	(0.0691)	(0.0688)	(0.0686)	(0.0695)	(0.0692)	(0.0692)	(0.0691)	(0.0691)	(0.0692)	(0.0691)
Hispanic	-0.145†	-0.157*	-0.150†	-0.157*	-0.157*	-0.156*	-0.150†	-0.160*	-0.160*	-0.158*	-0.157*	-0.157*	-0.159*	-0.158*
	(0.0770)	(0.0769)	(0.0769)	(0.0769)	(0.0769)	(0.0768)	(0.0773)	(0.0769)	(0.0770)	(0.0771)	(0.0769)	(0.0769)	(0.0770)	(0.0769)
Asian	0.315**	0.316**	0.315**	0.316**	0.316**	0.316**	0.314**	0.300**	0.312**	0.307**	0.316**	0.316**	0.313**	0.304**
	(0.0993)	(0.0996)	(0.0997)	(0.0997)	(0.0997)	(0.0996)	(0.0996)	(0.0995)	(0.0995)	(0.0996)	(0.0996)	(0.0996)	(0.100)	(0.0998)
[d]NI/AN	0.638*	0.686**	0.687**	0.685**	0.686**	0.683**	0.692**	0.620*	0.683**	0.685**	0.686**	0.686**	0.675*	0.692**
	(0.256)	(0.259)	(0.258)	(0.259)	(0.259)	(0.258)	(0.259)	(0.255)	(0.258)	(0.257)	(0.259)	(0.259)	(0.258)	(0.259)
[e]NHOPI	0.278	0.267	0.271	0.267	0.267	0.268	0.271	0.276	0.267	0.275	0.267	0.267	0.269	0.271
	(0.416)	(0.416)	(0.415)	(0.416)	(0.416)	(0.416)	(0.415)	(0.415)	(0.416)	(0.414)	(0.416)	(0.416)	(0.415)	(0.414)
Multi-Race/Other	0.256†	0.266†	0.268†	0.266†	0.267†	0.267†	0.267†	0.240	0.263†	0.251†	0.267†	0.267†	0.262†	0.264†
	(0.151)	(0.150)	(0.150)	(0.150)	(0.150)	(0.150)	(0.150)	(0.151)	(0.151)	(0.151)	(0.150)	(0.150)	(0.150)	(0.150)
Religious Salience	- 0.0503***	- 0.0504***	- 0.0506***	- 0.0504***	- 0.0504***	- 0.0504***	- 0.0506***	- 0.0507***	- 0.0508***	- 0.0507***	- 0.0504***	- 0.0503***	- 0.0504***	- 0.0508***

	(0.0110)	(0.0109)	(0.0110)	(0.0110)	(0.0110)	(0.0110)	(0.0110)	(0.0110)	(0.0110)	(0.0110)	(0.0110)	(0.0110)	(0.0110)	(0.0110)
Religious Attendance	-0.120***	-0.119***	-0.120***	-0.119***	-0.119***	-0.119***	-0.120***	-0.120***	-0.119***	-0.121***	-0.119***	-0.119***	-0.120***	-0.120***
	(0.0146)	(0.0147)	(0.0147)	(0.0147)	(0.0147)	(0.0147)	(0.0147)	(0.0146)	(0.0147)	(0.0147)	(0.0147)	(0.0147)	(0.0147)	(0.0146)
Cocaine	-0.103	-0.0844	-0.0802	-0.0845	-0.0845	-0.0838	-0.0822	-0.108	-0.0815	-0.0834	-0.0845	-0.0845	-0.0851	-0.0851
	(0.0675)	(0.0689)	(0.0688)	(0.0687)	(0.0687)	(0.0688)	(0.0688)	(0.0674)	(0.0687)	(0.0689)	(0.0687)	(0.0687)	(0.0688)	(0.0685)
Stimulant	0.642***	0.652***	0.651***	0.652***	0.652***	0.651***	0.650***	0.649***	0.655***	0.655***	0.651***	0.652***	0.654***	0.655***
	(0.0681)	(0.0682)	(0.0681)	(0.0682)	(0.0682)	(0.0682)	(0.0682)	(0.0683)	(0.0684)	(0.0681)	(0.0681)	(0.0682)	(0.0681)	(0.0681)
Sedative	0.855***	0.873***	0.874***	0.873***	0.873***	0.874***	0.875***	0.843***	0.870***	0.864***	0.873***	0.873***	0.876***	0.870***
	(0.0721)	(0.0725)	(0.0725)	(0.0725)	(0.0725)	(0.0723)	(0.0723)	(0.0728)	(0.0726)	(0.0730)	(0.0725)	(0.0725)	(0.0725)	(0.0730)
Tranquilizer	1.027***	1.026***	1.025***	1.026***	1.026***	1.025***	1.026***	1.030***	1.029***	1.029***	1.026***	1.026***	1.026***	1.026***
	(0.0552)	(0.0552)	(0.0553)	(0.0552)	(0.0552)	(0.0551)	(0.0552)	(0.0554)	(0.0551)	(0.0555)	(0.0552)	(0.0552)	(0.0551)	(0.0553)
Heroine	0.549***	0.626***	0.598***	0.627***	0.627***	0.622***	0.592***	0.510***	0.594***	0.563***	0.627***	0.628***	0.609***	0.570***
	(0.148)	(0.147)	(0.147)	(0.145)	(0.145)	(0.145)	(0.147)	(0.147)	(0.147)	(0.146)	(0.145)	(0.145)	(0.145)	(0.145)
Pain Killers	0.336***	0.341***	0.340***	0.341***	0.341***	0.341***	0.339***	0.341***	0.342***	0.346***	0.341***	0.341***	0.341***	0.341***
	(0.0612)	(0.0611)	(0.0611)	(0.0611)	(0.0611)	(0.0611)	(0.0612)	(0.0610)	(0.0609)	(0.0610)	(0.0611)	(0.0611)	(0.0611)	(0.0610)
Marijuana	0.416***	0.408***	0.410***	0.408***	0.408***	0.409***	0.410***	0.403***	0.405***	0.404***	0.409***	0.408***	0.405***	0.400***
	(0.0679)	(0.0679)	(0.0680)	(0.0679)	(0.0678)	(0.0680)	(0.0681)	(0.0676)	(0.0680)	(0.0680)	(0.0679)	(0.0679)	(0.0681)	(0.0676)
PCP	-0.0735	0.0501	0.0363	0.0505	0.0497	0.0473	0.0311	-0.104	0.0368	0.0214	0.0511	0.0496	0.0383	0.0146
	(0.179)	(0.179)	(0.178)	(0.179)	(0.179)	(0.178)	(0.179)	(0.178)	(0.179)	(0.179)	(0.179)	(0.179)	(0.179)	(0.177)
Inhalants	0.511***	0.531***	0.532***	0.531***	0.531***	0.532***	0.534***	0.519***	0.535***	0.536***	0.531***	0.531***	0.534***	0.539***
	(0.0732)	(0.0728)	(0.0729)	(0.0728)	(0.0728)	(0.0729)	(0.0730)	(0.0730)	(0.0726)	(0.0728)	(0.0728)	(0.0728)	(0.0727)	(0.0726)
Tobacco	0.409***	0.403***	0.405***	0.403***	0.403***	0.403***	0.405***	0.416***	0.404***	0.408***	0.403***	0.403***	0.406***	0.408***
	(0.0547)	(0.0547)	(0.0548)	(0.0547)	(0.0547)	(0.0547)	(0.0547)	(0.0548)	(0.0547)	(0.0548)	(0.0547)	(0.0547)	(0.0549)	(0.0547)
Age of 1st Alcohol Use	-0.0331	-0.0353	-0.0340	-0.0354	-0.0355	-0.0351	-0.0338	-0.0308	-0.0332	-0.0319	-0.0353	-0.0355	-0.0338	-0.0326
	(0.0270)	(0.0269)	(0.0269)	(0.0269)	(0.0269)	(0.0269)	(0.0270)	(0.0270)	(0.0268)	(0.0269)	(0.0269)	(0.0269)	(0.0270)	(0.0269)
Thrill Seeking	0.772***	0.775***	0.774***	0.775***	0.775***	0.775***	0.774***	0.773***	0.774***	0.775***	0.775***	0.775***	0.775***	0.775***
	(0.0332)	(0.0331)	(0.0331)	(0.0331)	(0.0331)	(0.0331)	(0.0330)	(0.0333)	(0.0331)	(0.0332)	(0.0331)	(0.0331)	(0.0331)	(0.0331)
[f]2009	-0.0565	-0.0552	-0.0557	-0.0552	-0.0553	-0.0554	-0.0547	-0.0600	-0.0553	-0.0579	-0.0552	-0.0553	-0.0571	-0.0578
	(0.119)	(0.119)	(0.119)	(0.119)	(0.119)	(0.119)	(0.119)	(0.118)	(0.119)	(0.119)	(0.119)	(0.119)	(0.119)	(0.119)
2010	0.0345	0.0341	0.0338	0.0340	0.0340	0.0338	0.0354	0.0325	0.0348	0.0337	0.0340	0.0341	0.0324	0.0331
	(0.116)	(0.116)	(0.116)	(0.116)	(0.116)	(0.117)	(0.116)	(0.116)	(0.116)	(0.116)	(0.116)	(0.116)	(0.116)	(0.116)
2011	0.0290	0.0315	0.0306	0.0315	0.0316	0.0313	0.0318	0.0272	0.0301	0.0302	0.0315	0.0316	0.0300	0.0313

	(0.116)	(0.116)	(0.116)	(0.116)	(0.116)	(0.117)	(0.116)	(0.115)	(0.116)	(0.116)	(0.116)	(0.116)	(0.116)	(0.116)
2012	0.134	0.136	0.135	0.136	0.136	0.135	0.136	0.133	0.136	0.135	0.136	0.136	0.136	0.136
	(0.115)	(0.116)	(0.116)	(0.116)	(0.116)	(0.116)	(0.116)	(0.115)	(0.116)	(0.116)	(0.116)	(0.116)	(0.116)	(0.116)
2013	0.266†	0.266†	0.267†	0.266†	0.266†	0.266†	0.267†	0.265†	0.266†	0.266†	0.266†	0.266†	0.263†	0.267†
	(0.138)	(0.138)	(0.138)	(0.138)	(0.138)	(0.138)	(0.138)	(0.137)	(0.138)	(0.137)	(0.138)	(0.138)	(0.138)	(0.137)
2014	0.0867	0.0824	0.0822	0.0824	0.0823	0.0821	0.0830	0.0804	0.0838	0.0769	0.0825	0.0819	0.0785	0.0786
	(0.104)	(0.104)	(0.104)	(0.104)	(0.104)	(0.104)	(0.104)	(0.103)	(0.104)	(0.104)	(0.104)	(0.104)	(0.104)	(0.104)
2015	-0.299*	-0.304*	-0.304*	-0.304*	-0.305*	-0.305*	-0.302*	-0.307**	-0.305*	-0.310**	-0.304*	-0.305*	-0.308**	-0.307**
	(0.117)	(0.117)	(0.117)	(0.117)	(0.117)	(0.117)	(0.117)	(0.117)	(0.117)	(0.117)	(0.117)	(0.117)	(0.117)	(0.116)
2016	-0.177	-0.184†	-0.183†	-0.184†	-0.184†	-0.184†	-0.182	-0.192†	-0.186†	-0.196†	-0.184†	-0.184†	-0.190†	-0.192†
	(0.110)	(0.110)	(0.110)	(0.110)	(0.110)	(0.110)	(0.110)	(0.109)	(0.110)	(0.110)	(0.110)	(0.110)	(0.110)	(0.109)
2017	-0.0331	-0.0416	-0.0420	-0.0417	-0.0418	-0.0419	-0.0403	-0.0449	-0.0436	-0.0528	-0.0417	-0.0419	-0.0476	-0.0475
	(0.118)	(0.118)	(0.118)	(0.118)	(0.118)	(0.118)	(0.118)	(0.117)	(0.117)	(0.117)	(0.118)	(0.118)	(0.118)	(0.117)
2018	0.252*	0.245*	0.245*	0.245*	0.244*	0.244*	0.246*	0.241*	0.243*	0.237†	0.245*	0.244*	0.241*	0.241*
	(0.122)	(0.121)	(0.121)	(0.121)	(0.121)	(0.121)	(0.121)	(0.121)	(0.121)	(0.121)	(0.121)	(0.121)	(0.121)	(0.121)
2019	0.555***	0.546***	0.546***	0.546***	0.546***	0.545***	0.547***	0.541***	0.543***	0.537***	0.546***	0.546***	0.539***	0.538***
	(0.126)	(0.126)	(0.126)	(0.126)	(0.126)	(0.126)	(0.126)	(0.126)	(0.126)	(0.126)	(0.126)	(0.126)	(0.126)	(0.126)
MDMA	0.143†	0.155	0.141†	0.147†	0.147†	0.146†	0.142†	0.181*	0.0216	0.162†	0.147†	0.147†	0.155†	0.164†
	(0.0835)	(0.119)	(0.0840)	(0.0843)	(0.0843)	(0.0844)	(0.0840)	(0.0828)	(0.0876)	(0.0837)	(0.0843)	(0.0843)	(0.0844)	(0.0834)
LCPU	0.0642							-0.405***						
	(0.0970)							(0.0748)						
Priv Hlth Ins	-0.904***	-0.977***	-0.936***	-0.979***	-0.979***	-0.973***	-0.930***	-0.962***	-0.972***	-0.968***	-0.979***	-0.979***	-0.980***	-0.968***
	(0.0649)	(0.0641)	(0.0659)	(0.0626)	(0.0623)	(0.0639)	(0.0621)	(0.0620)	(0.0621)	(0.0621)	(0.0624)	(0.0624)	(0.0622)	(0.0620)
Pub Hlth Ins	0.117*	0.117*	0.118*	0.117*	0.117*	0.118*	0.117*	-0.0343	0.0663	0.0216	0.118*	0.117*	0.0747	0.0153
	(0.0568)	(0.0569)	(0.0571)	(0.0570)	(0.0569)	(0.0569)	(0.0571)	(0.0629)	(0.0591)	(0.0608)	(0.0568)	(0.0570)	(0.0590)	(0.0614)
Oth Hlth Ins	-0.626***	-0.631***	-0.628***	-0.631***	-0.630***	-0.630***	-0.628***	-0.613***	-0.622***	-0.622***	-0.631***	-0.631***	-0.629***	-0.621***
	(0.141)	(0.141)	(0.141)	(0.141)	(0.141)	(0.141)	(0.141)	(0.141)	(0.140)	(0.141)	(0.141)	(0.141)	(0.141)	(0.141)
LCPU * Priv Hlth Ins	-0.378**													
	(0.114)													
Psilocybin		-0.296***	-0.0845	-0.296***	-0.296***	-0.296***	-0.293***		-0.288***	-0.478***	-0.296***	-0.296***	-0.295***	-0.281**
		(0.0839)	(0.120)	(0.0838)	(0.0839)	(0.0839)	(0.0837)		(0.0840)	(0.0871)	(0.0839)	(0.0839)	(0.0837)	(0.0834)
DMT		0.659	0.646	0.597	0.637	0.658	0.651		0.712	0.751	0.714	0.625	0.719	0.750
		(0.578)	(0.576)	(0.930)	(0.583)	(0.578)	(0.576)		(0.574)	(0.576)	(0.588)	(0.587)	(0.578)	(0.576)

Ayahuasca	1.397	1.410	1.410	2.705	1.399	1.411		1.403	1.409	1.468	0.889	1.405	1.407
	(1.673)	(1.676)	(1.691)	(2.150)	(1.673)	(1.676)		(1.675)	(1.663)	(1.731)	(1.942)	(1.675)	(1.663)
Pey/Mesc	-0.473***	-0.480***	-0.472***	-0.472***	-0.411†	-0.474***		-0.478***	-0.516***	-0.473***	-0.473***	-0.686***	-0.507***
	(0.124)	(0.124)	(0.124)	(0.124)	(0.221)	(0.124)		(0.124)	(0.123)	(0.124)	(0.124)	(0.142)	(0.123)
LSD	0.125	0.125	0.125	0.125	0.126	0.336**		0.124	0.120	0.125	0.125	0.123	-0.0657
	(0.0832)	(0.0832)	(0.0832)	(0.0832)	(0.0828)	(0.121)		(0.0831)	(0.0833)	(0.0832)	(0.0833)	(0.0833)	(0.0959)
MDMA * Priv Hlth Ins	-0.0134												
	(0.127)												
Psilocybin * Priv Hlth Ins		-0.318*											
		(0.125)											
DMT * Priv Hlth Ins			0.112										
			(1.041)										
Ayahuasca * Priv Hlth Ins				-2.006									
				(2.784)									
Pey/Mesc * Priv Hlth Ins					-0.103								
					(0.257)								
LSD * Priv Hlth Ins						-0.327*							
						(0.148)							
LCPU * Pub Hlth Ins							0.936***						
							(0.151)						
MDMA * Pub Hlth Ins								0.582***					
								(0.149)					
Psilocybin * Pub Hlth Ins									0.923***				
									(0.170)				
DMT * Pub Hlth Ins										-0.515			
										(1.824)			

Ayahuasca * Pub Hlth Ins												3.240		
												(2.899)		
Pey/Mesc * Pub Hlth Ins													0.719*	
													(0.275)	
LSD * Pub Hlth Ins														0.796***
														(0.169)
Constant	16.75***	16.77***	16.74***	16.77***	16.77***	16.76***	16.74***	16.79***	16.76***	16.76***	16.77***	16.77***	16.77***	16.76***
	(0.206)	(0.207)	(0.206)	(0.207)	(0.207)	(0.206)	(0.208)	(0.206)	(0.207)	(0.208)	(0.207)	(0.208)	(0.207)	(0.208)
Observations	158268	158268	158268	158268	158268	158268	158268	158268	158268	158268	158268	158268	158268	158268
R^2	0.154	0.155	0.155	0.155	0.155	0.155	0.155	0.155	0.155	0.155	0.155	0.155	0.155	0.155

Standard errors in parentheses
Source: 2008-2019 National Survey of Drug Use and Health, n=484,732
[a]Men serve as the reference category for Women
[b]Single serves as the reference category for Marital Statuses
[c]White serves as the reference category for race/ethnicity
[d]Native Indian or Alaskan Native
[e]Native Hawaiian or Pacific Islander
[f]2008 serves as the reference category for the survey year
† $p < .10$, * $p < .05$, ** $p < .01$, *** $p < .001$

Supplemental Table 5. Weighted Multivariate Ordinary Least Square Regression Predicting Psychological Distress Among Men with Interaction Terms (Psychedelics (x) Private or Public Health Insurance)

	Model 1	Model 2	Model 3	Model 4	Model 5	Model 6	Model 7	Model 8	Model 9	Model 10	Model 11	Model 12	Model 13	Model 14
Age	-0.376***	-0.374***	-0.374***	-0.374***	-0.374***	-0.374***	-0.374***	-0.373***	-0.372***	-0.371***	-0.374***	-0.374***	-0.373***	-0.372***
	(0.0187)	(0.0189)	(0.0189)	(0.0189)	(0.0189)	(0.0190)	(0.0190)	(0.0184)	(0.0190)	(0.0188)	(0.0189)	(0.0189)	(0.0188)	(0.0188)
Family Income	-0.286***	-0.287***	-0.287***	-0.287***	-0.287***	-0.287***	-0.287***	-0.286***	-0.287***	-0.286***	-0.287***	-0.287***	-0.286***	-0.286***
	(0.0207)	(0.0206)	(0.0207)	(0.0207)	(0.0207)	(0.0207)	(0.0207)	(0.0207)	(0.0207)	(0.0207)	(0.0207)	(0.0207)	(0.0207)	(0.0207)
Education Level	-0.301***	-0.298***	-0.299***	-0.298***	-0.298***	-0.298***	-0.299***	-0.305***	-0.300***	-0.302***	-0.298***	-0.298***	-0.300***	-0.302***
	(0.0427)	(0.0430)	(0.0430)	(0.0430)	(0.0430)	(0.0429)	(0.0429)	(0.0427)	(0.0431)	(0.0430)	(0.0430)	(0.0430)	(0.0432)	(0.0431)
[a]Married	-0.833***	-0.829***	-0.827***	-0.830***	-0.829***	-0.830***	-0.828***	-0.829***	-0.828***	-0.823***	-0.829***	-0.829***	-0.825***	-0.822***
	(0.106)	(0.105)	(0.106)	(0.105)	(0.105)	(0.106)	(0.106)	(0.106)	(0.106)	(0.106)	(0.105)	(0.105)	(0.105)	(0.106)
Widowed	-0.236	-0.232	-0.231	-0.233	-0.232	-0.233	-0.231	-0.214	-0.220	-0.204	-0.232	-0.232	-0.224	-0.213
	(0.261)	(0.260)	(0.260)	(0.260)	(0.260)	(0.260)	(0.260)	(0.262)	(0.260)	(0.262)	(0.260)	(0.260)	(0.260)	(0.261)
Divorced	0.138	0.146	0.147	0.146	0.147	0.148	0.146	0.133	0.146	0.140	0.147	0.147	0.147	0.144
	(0.148)	(0.147)	(0.148)	(0.148)	(0.148)	(0.148)	(0.148)	(0.148)	(0.148)	(0.148)	(0.148)	(0.147)	(0.148)	(0.148)
Children	0.192***	0.189***	0.189***	0.189***	0.188***	0.189***	0.189***	0.185***	0.184***	0.181***	0.188***	0.188***	0.187***	0.184***
	(0.0464)	(0.0460)	(0.0460)	(0.0460)	(0.0461)	(0.0461)	(0.0461)	(0.0470)	(0.0463)	(0.0463)	(0.0460)	(0.0461)	(0.0458)	(0.0468)
[b]Black	-0.357**	-0.366***	-0.357**	-0.363***	-0.363***	-0.365***	-0.360***	-0.362***	-0.364***	-0.355***	-0.363***	-0.364***	-0.363***	-0.358***
	(0.106)	(0.106)	(0.107)	(0.106)	(0.105)	(0.106)	(0.106)	(0.105)	(0.106)	(0.105)	(0.106)	(0.105)	(0.105)	(0.105)
Hispanic	-0.0524	-0.0609	-0.0543	-0.0584	-0.0589	-0.0603	-0.0559	-0.0660	-0.0631	-0.0632	-0.0590	-0.0588	-0.0605	-0.0608
	(0.118)	(0.117)	(0.116)	(0.117)	(0.117)	(0.117)	(0.117)	(0.118)	(0.117)	(0.117)	(0.117)	(0.117)	(0.117)	(0.117)
Asian	0.436*	0.438*	0.439*	0.439*	0.439*	0.438*	0.438*	0.426*	0.436*	0.432*	0.438*	0.438*	0.436*	0.431*
	(0.168)	(0.168)	(0.168)	(0.168)	(0.168)	(0.168)	(0.168)	(0.169)	(0.168)	(0.168)	(0.168)	(0.168)	(0.169)	(0.169)
[c]NI/AN	1.112*	1.161*	1.165*	1.165*	1.163*	1.165*	1.166*	1.103*	1.159*	1.171*	1.163*	1.163*	1.159*	1.168*
	(0.465)	(0.473)	(0.472)	(0.473)	(0.472)	(0.473)	(0.472)	(0.461)	(0.468)	(0.465)	(0.472)	(0.472)	(0.470)	(0.471)
[d]NHOPI	0.212	0.217	0.222	0.218	0.217	0.216	0.220	0.200	0.221	0.219	0.217	0.217	0.219	0.217
	(0.701)	(0.696)	(0.698)	(0.697)	(0.696)	(0.697)	(0.698)	(0.697)	(0.695)	(0.693)	(0.696)	(0.696)	(0.695)	(0.695)
Multi-Race/Other	0.277	0.284	0.284	0.283	0.286	0.283	0.283	0.264	0.283	0.262	0.282	0.284	0.280	0.280
	(0.207)	(0.207)	(0.207)	(0.207)	(0.207)	(0.207)	(0.207)	(0.207)	(0.208)	(0.207)	(0.207)	(0.207)	(0.207)	(0.207)
Religious Salience	-0.0589***	-0.0584***	-0.0588***	-0.0586***	-0.0586***	-0.0585***	-0.0587***	-0.0593***	-0.0588***	-0.0590***	-0.0585***	-0.0585***	-0.0586***	-0.0591***
	(0.0164)	(0.0163)	(0.0163)	(0.0163)	(0.0163)	(0.0163)	(0.0163)	(0.0164)	(0.0163)	(0.0163)	(0.0163)	(0.0163)	(0.0163)	(0.0163)
Religious Attendance	-0.0693**	-0.0690**	-0.0695**	-0.0692**	-0.0692**	-0.0691**	-0.0695**	-0.0693**	-0.0693**	-0.0704**	-0.0692**	-0.0692**	-0.0697**	-0.0697**
	(0.0220)	(0.0220)	(0.0219)	(0.0219)	(0.0219)	(0.0219)	(0.0219)	(0.0220)	(0.0219)	(0.0219)	(0.0219)	(0.0219)	(0.0220)	(0.0219)

Cocaine	-0.210*	-0.218*	-0.213*	-0.216*	-0.216*	-0.217*	-0.215*	-0.216*	-0.213*	-0.215*	-0.216*	-0.215*	-0.217*	-0.217*
	(0.104)	(0.107)	(0.107)	(0.107)	(0.107)	(0.107)	(0.107)	(0.104)	(0.107)	(0.107)	(0.107)	(0.107)	(0.107)	(0.107)
Stimulant	0.667***	0.665***	0.665***	0.666***	0.666***	0.666***	0.665***	0.673***	0.669***	0.670***	0.667***	0.666***	0.668***	0.670***
	(0.106)	(0.106)	(0.106)	(0.106)	(0.106)	(0.106)	(0.106)	(0.106)	(0.106)	(0.106)	(0.106)	(0.106)	(0.106)	(0.106)
Sedative	0.513***	0.545***	0.545***	0.545***	0.546***	0.544***	0.546***	0.498***	0.541***	0.529***	0.546***	0.545***	0.545***	0.539***
	(0.111)	(0.112)	(0.112)	(0.112)	(0.112)	(0.112)	(0.112)	(0.111)	(0.111)	(0.111)	(0.112)	(0.112)	(0.112)	(0.112)
Tranquilizer	0.942***	0.932***	0.933***	0.933***	0.934***	0.933***	0.933***	0.944***	0.936***	0.937***	0.933***	0.934***	0.933***	0.932***
	(0.0909)	(0.0895)	(0.0897)	(0.0896)	(0.0896)	(0.0896)	(0.0896)	(0.0911)	(0.0893)	(0.0898)	(0.0896)	(0.0894)	(0.0896)	(0.0897)
Heroine	0.782***	0.826***	0.805***	0.817***	0.818***	0.821***	0.808***	0.764***	0.796***	0.775***	0.819***	0.818***	0.809***	0.789***
	(0.187)	(0.188)	(0.187)	(0.186)	(0.186)	(0.186)	(0.187)	(0.189)	(0.189)	(0.189)	(0.186)	(0.186)	(0.187)	(0.187)
Pain Killers	0.316***	0.315***	0.315***	0.315***	0.314***	0.315***	0.315***	0.320***	0.317***	0.321***	0.315***	0.314***	0.315***	0.315***
	(0.0873)	(0.0875)	(0.0874)	(0.0875)	(0.0873)	(0.0874)	(0.0875)	(0.0872)	(0.0873)	(0.0876)	(0.0875)	(0.0874)	(0.0875)	(0.0875)
Marijuana	0.418***	0.409***	0.410***	0.409***	0.409***	0.409***	0.410***	0.404***	0.406***	0.401***	0.409***	0.409***	0.405***	0.400***
	(0.106)	(0.105)	(0.105)	(0.105)	(0.105)	(0.105)	(0.105)	(0.106)	(0.105)	(0.105)	(0.105)	(0.105)	(0.105)	(0.105)
PCP	-0.480*	-0.369	-0.383†	-0.375†	-0.376†	-0.373	-0.382†	-0.501*	-0.387†	-0.401†	-0.377†	-0.375†	-0.385†	-0.399†
	(0.224)	(0.225)	(0.226)	(0.225)	(0.225)	(0.225)	(0.226)	(0.224)	(0.226)	(0.226)	(0.226)	(0.226)	(0.226)	(0.226)
Inhalants	0.630***	0.627***	0.630***	0.629***	0.630***	0.629***	0.630***	0.635***	0.632***	0.633***	0.629***	0.629***	0.631***	0.634***
	(0.106)	(0.106)	(0.107)	(0.107)	(0.106)	(0.106)	(0.107)	(0.107)	(0.107)	(0.107)	(0.106)	(0.106)	(0.106)	(0.107)
Tobacco	-0.00666	-0.0117	-0.0113	-0.0119	-0.0121	-0.0119	-0.0116	0.00290	-0.00996	-0.00285	-0.0120	-0.0119	-0.00906	-0.00648
	(0.0933)	(0.0927)	(0.0926)	(0.0927)	(0.0927)	(0.0927)	(0.0927)	(0.0935)	(0.0926)	(0.0928)	(0.0927)	(0.0927)	(0.0931)	(0.0930)
Age of 1st Alcohol Use	0.0122	0.0112	0.0123	0.0116	0.0113	0.0114	0.0121	0.0129	0.0130	0.0141	0.0115	0.0115	0.0124	0.0128
	(0.0409)	(0.0408)	(0.0409)	(0.0409)	(0.0408)	(0.0409)	(0.0409)	(0.0409)	(0.0408)	(0.0408)	(0.0409)	(0.0409)	(0.0409)	(0.0409)
Thrill Seeking	0.647***	0.649***	0.648***	0.648***	0.648***	0.649***	0.648***	0.648***	0.647***	0.648***	0.649***	0.648***	0.648***	0.649***
	(0.0515)	(0.0516)	(0.0514)	(0.0513)	(0.0514)	(0.0514)	(0.0514)	(0.0517)	(0.0515)	(0.0517)	(0.0514)	(0.0514)	(0.0515)	(0.0515)
[e]2009	0.176	0.182	0.183	0.183	0.182	0.183	0.184	0.172	0.183	0.179	0.183	0.183	0.182	0.181
	(0.221)	(0.221)	(0.222)	(0.221)	(0.221)	(0.221)	(0.222)	(0.221)	(0.221)	(0.222)	(0.221)	(0.221)	(0.222)	(0.222)
2010	0.123	0.120	0.121	0.121	0.120	0.121	0.121	0.116	0.121	0.115	0.121	0.121	0.119	0.116
	(0.155)	(0.155)	(0.155)	(0.155)	(0.155)	(0.156)	(0.155)	(0.155)	(0.155)	(0.155)	(0.155)	(0.155)	(0.155)	(0.155)
2011	0.250	0.250	0.249	0.249	0.250	0.250	0.249	0.242	0.247	0.242	0.250	0.250	0.248	0.245
	(0.174)	(0.175)	(0.175)	(0.175)	(0.175)	(0.175)	(0.175)	(0.174)	(0.175)	(0.176)	(0.175)	(0.175)	(0.175)	(0.175)
2012	0.404*	0.401*	0.402*	0.402*	0.402*	0.402*	0.402*	0.402*	0.401*	0.400*	0.402*	0.402*	0.403*	0.401*
	(0.168)	(0.168)	(0.169)	(0.169)	(0.169)	(0.168)	(0.169)	(0.169)	(0.169)	(0.169)	(0.168)	(0.169)	(0.169)	(0.169)

2013	0.489*	0.489*	0.490*	0.489*	0.488*	0.490*	0.489*	0.481*	0.488*	0.484*	0.488*	0.489*	0.485*	0.485*
	(0.200)	(0.200)	(0.200)	(0.200)	(0.200)	(0.201)	(0.200)	(0.199)	(0.200)	(0.200)	(0.200)	(0.200)	(0.201)	(0.199)
2014	0.183	0.172	0.173	0.173	0.172	0.174	0.173	0.174	0.175	0.163	0.173	0.172	0.168	0.167
	(0.183)	(0.183)	(0.183)	(0.183)	(0.183)	(0.183)	(0.183)	(0.183)	(0.183)	(0.184)	(0.183)	(0.183)	(0.184)	(0.183)
2015	-0.0761	-0.0727	-0.0717	-0.0717	-0.0724	-0.0708	-0.0713	-0.0825	-0.0715	-0.0779	-0.0717	-0.0719	-0.0744	-0.0743
	(0.170)	(0.170)	(0.170)	(0.170)	(0.170)	(0.170)	(0.170)	(0.169)	(0.170)	(0.170)	(0.170)	(0.170)	(0.170)	(0.169)
2016	0.187	0.190	0.189	0.190	0.189	0.190	0.190	0.174	0.187	0.175	0.190	0.189	0.185	0.182
	(0.162)	(0.162)	(0.162)	(0.162)	(0.162)	(0.162)	(0.162)	(0.161)	(0.162)	(0.162)	(0.162)	(0.162)	(0.162)	(0.161)
2017	0.373*	0.368*	0.368*	0.368*	0.368*	0.369*	0.368*	0.361*	0.365*	0.354*	0.369*	0.368*	0.364*	0.361*
	(0.171)	(0.170)	(0.170)	(0.170)	(0.170)	(0.171)	(0.170)	(0.171)	(0.170)	(0.171)	(0.170)	(0.170)	(0.170)	(0.170)
2018	0.734***	0.733***	0.732***	0.733***	0.732***	0.733***	0.732***	0.725***	0.729***	0.721***	0.733***	0.732***	0.732***	0.728***
	(0.183)	(0.183)	(0.183)	(0.183)	(0.183)	(0.183)	(0.183)	(0.183)	(0.183)	(0.183)	(0.183)	(0.183)	(0.183)	(0.182)
2019	0.988***	0.981***	0.981***	0.981***	0.981***	0.982***	0.982***	0.976***	0.977***	0.969***	0.982***	0.981***	0.978***	0.974***
	(0.190)	(0.189)	(0.190)	(0.190)	(0.190)	(0.190)	(0.189)	(0.190)	(0.190)	(0.190)	(0.189)	(0.190)	(0.190)	(0.189)
MDMA	0.202†	0.0759	0.159	0.162	0.163	0.163	0.161	0.232*	0.0776	0.181	0.162	0.163	0.169	0.177
	(0.115)	(0.169)	(0.119)	(0.119)	(0.119)	(0.119)	(0.119)	(0.116)	(0.124)	(0.119)	(0.119)	(0.119)	(0.119)	(0.119)
LCPU	-0.00468							-0.280*						
	(0.162)							(0.125)						
Priv Hlth Ins	-0.758***	-0.838***	-0.786***	-0.813***	-0.815***	-0.824***	-0.797***	-0.807***	-0.812***	-0.807***	-0.816***	-0.816***	-0.817***	-0.811***
	(0.101)	(0.0984)	(0.102)	(0.0908)	(0.0897)	(0.0905)	(0.0972)	(0.0888)	(0.0898)	(0.0894)	(0.0898)	(0.0898)	(0.0897)	(0.0890)
Pub Hlth Ins	0.398***	0.400***	0.402***	0.402***	0.401***	0.401***	0.401***	0.263*	0.352***	0.276**	0.398***	0.400***	0.362***	0.308**
	(0.0953)	(0.0951)	(0.0952)	(0.0950)	(0.0951)	(0.0950)	(0.0951)	(0.111)	(0.0995)	(0.105)	(0.0950)	(0.0951)	(0.102)	(0.109)
Oth Hlth Ins	-0.486*	-0.493*	-0.488*	-0.492*	-0.491*	-0.493*	-0.490*	-0.484*	-0.487*	-0.487*	-0.492*	-0.492*	-0.491*	-0.487*
	(0.216)	(0.217)	(0.217)	(0.217)	(0.217)	(0.217)	(0.218)	(0.215)	(0.216)	(0.216)	(0.217)	(0.217)	(0.217)	(0.216)
LCPU * Priv Hlth Ins	-0.216													
	(0.155)													
Psilocybin		-0.198	-0.0993	-0.197	-0.197	-0.197	-0.196		-0.194	-0.345*	-0.197	-0.197	-0.196	-0.186
		(0.133)	(0.200)	(0.133)	(0.133)	(0.133)	(0.133)		(0.133)	(0.133)	(0.133)	(0.133)	(0.133)	(0.133)
DMT		1.205†	1.200†	1.615	1.160†	1.206†	1.203†		1.237†	1.278*	1.061	1.174†	1.245†	1.259†
		(0.642)	(0.637)	(0.998)	(0.647)	(0.640)	(0.639)		(0.635)	(0.642)	(0.668)	(0.648)	(0.642)	(0.641)
Ayahuasca		2.009	2.030	1.932	5.834	2.014	2.025		2.039	2.047	1.865	1.348	2.028	2.042
		(2.520)	(2.518)	(2.538)	(3.853)	(2.521)	(2.519)		(2.522)	(2.498)	(2.509)	(2.777)	(2.510)	(2.505)
Pey/Mesc		-0.484*	-0.490*	-0.486*	-0.486*	-0.535†	-0.486*		-0.491*	-0.530**	-0.483*	-0.486*	-0.605**	-0.512**

	(0.188)	(0.188)	(0.188)	(0.188)	(0.276)	(0.188)		(0.188)	(0.186)	(0.187)	(0.188)	(0.203)	(0.187)
LSD	0.219†	0.221†	0.220†	0.221†	0.219†	0.280		0.218†	0.217	0.220†	0.220†	0.219†	0.106
	(0.131)	(0.131)	(0.131)	(0.131)	(0.131)	(0.185)		(0.131)	(0.131)	(0.131)	(0.131)	(0.131)	(0.144)
MDMA * Priv Hlth Ins	0.146												
	(0.187)												
Psilocybin * Priv Hlth Ins		-0.149											
		(0.181)											
DMT * Priv Hlth Ins			-0.732										
			(1.087)										
Ayahuasca * Priv Hlth Ins				-5.612									
				(4.770)									
Pey/Mesc * Priv Hlth Ins					0.0853								
					(0.281)								
LSD * Priv Hlth Ins						-0.0935							
						(0.174)							
LCPU * Pub Hlth Ins							0.585						
							(0.307)						
MDMA * Pub Hlth Ins								0.461					
								(0.280)					
Psilocybin * Pub Hlth Ins									0.785				
									(0.438)				
DMT * Pub Hlth Ins										1.501			
										(1.196)			
Ayahuasca * Pub Hlth Ins											5.641†		
											(2.874)		
Pey/Mesc * Pub												0.401	

Hlth Ins														
													(0.320)	
LSD * Pub Hlth Ins														0.494†
														(0.268)
Constant	15.59***	15.60***	15.57***	15.59***	15.59***	15.59***	15.58***	15.63***	15.58***	15.58***	15.59***	15.59***	15.59***	15.59***
	(0.369)	(0.375)	(0.375)	(0.376)	(0.375)	(0.374)	(0.377)	(0.370)	(0.376)	(0.376)	(0.376)	(0.376)	(0.376)	(0.375)
Observations	58652	58652	58652	58652	58652	58652	58652	58652	58652	58652	58652	58652	58652	58652
R^2	0.136	0.136	0.136	0.136	0.136	0.136	0.136	0.136	0.136	0.137	0.136	0.136	0.136	0.136

Standard errors in parentheses

Source: 2008-2019 National Survey of Drug Use and Health, n=484,732

[a]Single serves as the reference category for Marital Statuses

[b]White serves as the reference category for race/ethnicity

[c]Native Indian or Alaskan Native

[d]Native Hawaiian or Pacific Islander

[e]2008 serves as the reference category for the survey year

† $p < .10$, * $p < .05$, ** $p < .01$, *** $p < .001$

Supplemental Table 6. Weighted Multivariate Ordinary Least Square Regression Predicting Psychological Distress Among Women with Interaction Terms (Psychedelics (x) Private or Public Health Insurance)

	Model 1	Model 2	Model 3	Model 4	Model 5	Model 6	Model 7	Model 8	Model 9	Model 10	Model 11	Model 12	Model 13	Model 14
Age	-0.515***	-0.512***	-0.513***	-0.512***	-0.512***	-0.513***	-0.513***	-0.511***	-0.511***	-0.510***	-0.512***	-0.512***	-0.511***	-0.510***
	(0.0133)	(0.0134)	(0.0133)	(0.0133)	(0.0133)	(0.0134)	(0.0133)	(0.0133)	(0.0133)	(0.0133)	(0.0133)	(0.0133)	(0.0133)	(0.0133)
Family Income	-0.286***	-0.287***	-0.287***	-0.287***	-0.287***	-0.286***	-0.286***	-0.286***	-0.288***	-0.287***	-0.287***	-0.287***	-0.286***	-0.287***
	(0.0168)	(0.0169)	(0.0168)	(0.0168)	(0.0168)	(0.0168)	(0.0169)	(0.0169)	(0.0169)	(0.0169)	(0.0168)	(0.0168)	(0.0169)	(0.0169)
Education Level	-0.330***	-0.325***	-0.326***	-0.325***	-0.326***	-0.326***	-0.328***	-0.334***	-0.326***	-0.328***	-0.325***	-0.325***	-0.328***	-0.330***
	(0.0297)	(0.0301)	(0.0301)	(0.0301)	(0.0301)	(0.0300)	(0.0301)	(0.0297)	(0.0301)	(0.0301)	(0.0301)	(0.0301)	(0.0302)	(0.0301)
[a]Married	-0.413***	-0.418***	-0.415***	-0.419***	-0.419***	-0.418***	-0.414***	-0.415***	-0.419***	-0.417***	-0.419***	-0.419***	-0.418***	-0.418***
	(0.0759)	(0.0759)	(0.0758)	(0.0759)	(0.0759)	(0.0758)	(0.0758)	(0.0760)	(0.0759)	(0.0759)	(0.0758)	(0.0759)	(0.0757)	(0.0760)
Widowed	-0.461**	-0.472**	-0.469**	-0.474**	-0.473**	-0.473**	-0.464**	-0.435**	-0.464**	-0.458**	-0.474**	-0.473**	-0.466**	-0.451**
	(0.149)	(0.150)	(0.149)	(0.150)	(0.150)	(0.150)	(0.149)	(0.149)	(0.149)	(0.149)	(0.150)	(0.150)	(0.150)	(0.150)
Divorced	0.404***	0.408***	0.405***	0.407***	0.408***	0.406***	0.404***	0.393***	0.408***	0.401***	0.407***	0.408***	0.402***	0.397***
	(0.107)	(0.108)	(0.108)	(0.108)	(0.108)	(0.108)	(0.108)	(0.108)	(0.108)	(0.109)	(0.108)	(0.108)	(0.108)	(0.108)
Children	0.278***	0.273***	0.274***	0.273***	0.273***	0.274***	0.274***	0.271***	0.269***	0.270***	0.274***	0.273***	0.273***	0.269***
	(0.0272)	(0.0272)	(0.0272)	(0.0271)	(0.0271)	(0.0272)	(0.0272)	(0.0273)	(0.0274)	(0.0272)	(0.0271)	(0.0271)	(0.0271)	(0.0273)
[b]Black	-0.612***	-0.630***	-0.620***	-0.631***	-0.631***	-0.628***	-0.619***	-0.617***	-0.632***	-0.625***	-0.632***	-0.631***	-0.628***	-0.625***
	(0.0985)	(0.0981)	(0.0982)	(0.0982)	(0.0982)	(0.0981)	(0.0980)	(0.0988)	(0.0982)	(0.0983)	(0.0983)	(0.0982)	(0.0982)	(0.0983)
Hispanic	-0.184†	-0.195*	-0.188†	-0.198*	-0.197*	-0.195*	-0.187†	-0.199*	-0.198*	-0.196*	-0.198*	-0.197*	-0.198*	-0.197*
	(0.0959)	(0.0956)	(0.0958)	(0.0956)	(0.0956)	(0.0957)	(0.0959)	(0.0958)	(0.0957)	(0.0958)	(0.0956)	(0.0956)	(0.0956)	(0.0957)
Asian	0.206	0.211	0.208	0.212	0.212	0.210	0.206	0.194	0.208	0.204	0.212	0.212	0.210	0.200
	(0.131)	(0.131)	(0.132)	(0.131)	(0.131)	(0.131)	(0.131)	(0.131)	(0.131)	(0.131)	(0.131)	(0.131)	(0.132)	(0.132)
[c]NI/AN	0.355	0.405	0.402	0.402	0.404	0.390	0.408	0.337	0.402	0.397	0.402	0.404	0.386	0.409
	(0.361)	(0.366)	(0.366)	(0.367)	(0.367)	(0.366)	(0.366)	(0.361)	(0.367)	(0.366)	(0.367)	(0.367)	(0.366)	(0.366)
[d]NHOPI	0.276	0.263	0.264	0.262	0.262	0.264	0.264	0.284	0.258	0.272	0.262	0.262	0.263	0.269
	(0.453)	(0.456)	(0.454)	(0.455)	(0.455)	(0.455)	(0.453)	(0.452)	(0.456)	(0.453)	(0.455)	(0.455)	(0.455)	(0.452)
Multi-Race/Other	0.231	0.241	0.245	0.241	0.241	0.241	0.244	0.218	0.237	0.233	0.242	0.241	0.238	0.241
	(0.203)	(0.204)	(0.203)	(0.204)	(0.204)	(0.203)	(0.204)	(0.204)	(0.204)	(0.204)	(0.204)	(0.204)	(0.204)	(0.204)
Religious Salience	-0.0466**	-0.0469**	-0.0469**	-0.0468**	-0.0468**	-0.0469**	-0.0470**	-0.0468**	-0.0472**	-0.0470**	-0.0468**	-0.0468**	-0.0468**	-0.0469**
	(0.0150)	(0.0150)	(0.0150)	(0.0150)	(0.0150)	(0.0150)	(0.0150)	(0.0150)	(0.0150)	(0.0150)	(0.0150)	(0.0150)	(0.0150)	(0.0150)
Religious Attendance	-0.139***	-0.138***	-0.139***	-0.138***	-0.138***	-0.138***	-0.140***	-0.140***	-0.138***	-0.139***	-0.138***	-0.138***	-0.138***	-0.139***
	(0.0196)	(0.0196)	(0.0196)	(0.0197)	(0.0197)	(0.0197)	(0.0196)	(0.0196)	(0.0197)	(0.0197)	(0.0197)	(0.0197)	(0.0197)	(0.0196)

Cocaine	-0.0386	-0.0110	-0.00657	-0.0125	-0.0121	-0.0102	-0.0112	-0.0400	-0.00892	-0.0103	-0.0124	-0.0120	-0.0105	-0.0112
	(0.0864)	(0.0873)	(0.0875)	(0.0875)	(0.0874)	(0.0878)	(0.0873)	(0.0866)	(0.0875)	(0.0875)	(0.0873)	(0.0874)	(0.0874)	(0.0873)
Stimulant	0.650***	0.662***	0.658***	0.661***	0.661***	0.660***	0.659***	0.657***	0.663***	0.663***	0.660***	0.661***	0.661***	0.662***
	(0.0853)	(0.0855)	(0.0856)	(0.0855)	(0.0856)	(0.0856)	(0.0854)	(0.0854)	(0.0855)	(0.0857)	(0.0853)	(0.0856)	(0.0859)	(0.0858)
Sedative	1.058***	1.066***	1.068***	1.064***	1.066***	1.068***	1.069***	1.053***	1.064***	1.065***	1.066***	1.066***	1.072***	1.068***
	(0.0998)	(0.0998)	(0.0997)	(0.0995)	(0.0998)	(0.0996)	(0.0995)	(0.100)	(0.0999)	(0.100)	(0.0995)	(0.0998)	(0.1000)	(0.100)
Tranquilizer	1.093***	1.095***	1.094***	1.096***	1.095***	1.094***	1.095***	1.098***	1.097***	1.097***	1.095***	1.095***	1.096***	1.098***
	(0.0808)	(0.0804)	(0.0805)	(0.0805)	(0.0804)	(0.0805)	(0.0805)	(0.0808)	(0.0805)	(0.0807)	(0.0805)	(0.0804)	(0.0804)	(0.0805)
Heroine	0.368	0.459*	0.420†	0.480*	0.474*	0.461*	0.413†	0.321	0.437†	0.403†	0.483*	0.475*	0.451*	0.398†
	(0.226)	(0.225)	(0.224)	(0.225)	(0.225)	(0.223)	(0.224)	(0.229)	(0.226)	(0.225)	(0.224)	(0.225)	(0.223)	(0.226)
Pain Killers	0.381***	0.387***	0.386***	0.389***	0.388***	0.388***	0.383***	0.387***	0.389***	0.391***	0.389***	0.389***	0.389***	0.388***
	(0.0764)	(0.0760)	(0.0762)	(0.0762)	(0.0763)	(0.0762)	(0.0762)	(0.0760)	(0.0762)	(0.0760)	(0.0762)	(0.0762)	(0.0760)	(0.0759)
Marijuana	0.408***	0.402***	0.405***	0.402***	0.402***	0.403***	0.405***	0.398***	0.400***	0.400***	0.403***	0.402***	0.400***	0.397***
	(0.0779)	(0.0778)	(0.0781)	(0.0778)	(0.0778)	(0.0778)	(0.0780)	(0.0774)	(0.0776)	(0.0777)	(0.0777)	(0.0778)	(0.0777)	(0.0775)
PCP	0.416†	0.528*	0.512*	0.536*	0.534*	0.524*	0.505*	0.391†	0.521*	0.509*	0.545*	0.532*	0.526*	0.496*
	(0.219)	(0.227)	(0.226)	(0.227)	(0.227)	(0.226)	(0.225)	(0.220)	(0.227)	(0.227)	(0.225)	(0.227)	(0.227)	(0.227)
Inhalants	0.468***	0.493***	0.490***	0.491***	0.493***	0.493***	0.494***	0.474***	0.494***	0.496***	0.490***	0.492***	0.494***	0.498***
	(0.106)	(0.105)	(0.105)	(0.104)	(0.105)	(0.105)	(0.105)	(0.105)	(0.105)	(0.104)	(0.104)	(0.105)	(0.104)	(0.104)
Tobacco	0.552***	0.548***	0.551***	0.548***	0.548***	0.549***	0.551***	0.555***	0.549***	0.551***	0.548***	0.548***	0.550***	0.552***
	(0.0642)	(0.0643)	(0.0644)	(0.0643)	(0.0642)	(0.0643)	(0.0642)	(0.0641)	(0.0642)	(0.0642)	(0.0643)	(0.0642)	(0.0643)	(0.0640)
Age of 1st Alcohol Use	-0.0343	-0.0368	-0.0355	-0.0372	-0.0372	-0.0364	-0.0349	-0.0328	-0.0354	-0.0350	-0.0369	-0.0374	-0.0360	-0.0349
	(0.0348)	(0.0346)	(0.0347)	(0.0346)	(0.0346)	(0.0347)	(0.0347)	(0.0346)	(0.0345)	(0.0346)	(0.0346)	(0.0346)	(0.0346)	(0.0345)
Thrill Seeking	0.906***	0.910***	0.910***	0.911***	0.911***	0.910***	0.909***	0.906***	0.909***	0.910***	0.910***	0.911***	0.911***	0.909***
	(0.0436)	(0.0435)	(0.0435)	(0.0435)	(0.0435)	(0.0436)	(0.0434)	(0.0438)	(0.0435)	(0.0436)	(0.0434)	(0.0435)	(0.0435)	(0.0435)
[e]2009	-0.185	-0.188	-0.188	-0.188	-0.188	-0.188	-0.188	-0.187	-0.188	-0.189	-0.187	-0.188	-0.190	-0.190
	(0.161)	(0.161)	(0.160)	(0.161)	(0.161)	(0.161)	(0.161)	(0.159)	(0.160)	(0.160)	(0.161)	(0.160)	(0.160)	(0.160)
2010	-0.0142	-0.0154	-0.0153	-0.0156	-0.0158	-0.0152	-0.0128	-0.0108	-0.0148	-0.0126	-0.0160	-0.0159	-0.0168	-0.0120
	(0.154)	(0.154)	(0.154)	(0.154)	(0.154)	(0.154)	(0.154)	(0.154)	(0.154)	(0.154)	(0.154)	(0.154)	(0.153)	(0.153)
2011	-0.0857	-0.0857	-0.0854	-0.0870	-0.0854	-0.0850	-0.0823	-0.0811	-0.0859	-0.0818	-0.0852	-0.0853	-0.0859	-0.0812
	(0.152)	(0.152)	(0.152)	(0.153)	(0.153)	(0.153)	(0.152)	(0.152)	(0.152)	(0.153)	(0.153)	(0.153)	(0.152)	(0.152)
2012	-0.0260	-0.0227	-0.0250	-0.0244	-0.0237	-0.0247	-0.0232	-0.0249	-0.0237	-0.0238	-0.0244	-0.0235	-0.0243	-0.0235
	(0.151)	(0.152)	(0.152)	(0.152)	(0.152)	(0.152)	(0.152)	(0.151)	(0.152)	(0.152)	(0.152)	(0.152)	(0.152)	(0.152)
2013	0.144	0.141	0.141	0.140	0.140	0.141	0.144	0.148	0.141	0.143	0.140	0.141	0.140	0.145

	(0.181)	(0.181)	(0.181)	(0.181)	(0.181)	(0.181)	(0.181)	(0.181)	(0.181)	(0.180)	(0.181)	(0.181)	(0.180)	(0.180)
2014	0.0375	0.0360	0.0342	0.0363	0.0346	0.0349	0.0374	0.0353	0.0352	0.0330	0.0365	0.0346	0.0334	0.0345
	(0.142)	(0.142)	(0.142)	(0.142)	(0.142)	(0.142)	(0.142)	(0.142)	(0.142)	(0.142)	(0.142)	(0.142)	(0.142)	(0.142)
2015	-0.480**	-0.490**	-0.489**	-0.491**	-0.491**	-0.490**	-0.486**	-0.489**	-0.492**	-0.495**	-0.491**	-0.491**	-0.494**	-0.493**
	(0.166)	(0.165)	(0.165)	(0.165)	(0.165)	(0.165)	(0.165)	(0.166)	(0.165)	(0.165)	(0.165)	(0.165)	(0.165)	(0.165)
2016	-0.454**	-0.465**	-0.463**	-0.467**	-0.467**	-0.466**	-0.461**	-0.469**	-0.468**	-0.475**	-0.467**	-0.467**	-0.475**	-0.474**
	(0.154)	(0.154)	(0.154)	(0.154)	(0.154)	(0.153)	(0.153)	(0.153)	(0.154)	(0.153)	(0.154)	(0.154)	(0.153)	(0.153)
2017	-0.315†	-0.323*	-0.325*	-0.325*	-0.325*	-0.324*	-0.320*	-0.325*	-0.326*	-0.332*	-0.325*	-0.325*	-0.332*	-0.328*
	(0.162)	(0.161)	(0.161)	(0.161)	(0.161)	(0.161)	(0.161)	(0.162)	(0.161)	(0.161)	(0.161)	(0.161)	(0.161)	(0.161)
2018	-0.0961	-0.107	-0.106	-0.109	-0.109	-0.107	-0.102	-0.108	-0.108	-0.112	-0.109	-0.109	-0.114	-0.111
	(0.162)	(0.161)	(0.161)	(0.161)	(0.161)	(0.161)	(0.161)	(0.162)	(0.161)	(0.161)	(0.161)	(0.161)	(0.161)	(0.161)
2019	0.234	0.225	0.227	0.223	0.223	0.222	0.226	0.221	0.222	0.219	0.223	0.223	0.215	0.217
	(0.159)	(0.158)	(0.158)	(0.158)	(0.158)	(0.158)	(0.158)	(0.159)	(0.158)	(0.158)	(0.158)	(0.158)	(0.158)	(0.157)
MDMA	0.102	0.232	0.115	0.123	0.124	0.121	0.113	0.137	-0.00166	0.132	0.123	0.124	0.129	0.135
	(0.113)	(0.155)	(0.113)	(0.113)	(0.113)	(0.114)	(0.113)	(0.112)	(0.117)	(0.113)	(0.113)	(0.113)	(0.113)	(0.112)
LCPU	0.244							-0.411***						
	(0.150)							(0.113)						
Priv Hlth Ins	-0.970***	-1.040***	-1.002***	-1.060***	-1.058***	-1.040***	-0.989***	-1.036***	-1.049***	-1.046***	-1.057***	-1.057***	-1.057***	-1.043***
	(0.0783)	(0.0797)	(0.0784)	(0.0768)	(0.0767)	(0.0777)	(0.0763)	(0.0768)	(0.0764)	(0.0767)	(0.0768)	(0.0767)	(0.0767)	(0.0770)
Pub Hlth Ins	-0.0726	-0.0707	-0.0718	-0.0699	-0.0692	-0.0683	-0.0740	-0.198*	-0.109	-0.131	-0.0669	-0.0693	-0.104	-0.154†
	(0.0773)	(0.0777)	(0.0778)	(0.0778)	(0.0778)	(0.0779)	(0.0776)	(0.0786)	(0.0805)	(0.0790)	(0.0778)	(0.0778)	(0.0768)	(0.0784)
Oth Hlth Ins	-0.738***	-0.737***	-0.736***	-0.734***	-0.734***	-0.733***	-0.735***	-0.710***	-0.725***	-0.724***	-0.734***	-0.734***	-0.732***	-0.722***
	(0.181)	(0.180)	(0.181)	(0.180)	(0.181)	(0.180)	(0.180)	(0.181)	(0.180)	(0.180)	(0.180)	(0.180)	(0.181)	(0.181)
LCPU * Priv Hlth Ins	-0.597**													
	(0.182)													
Psilocybin		-0.230†	0.174	-0.233†	-0.234†	-0.235†	-0.230†		-0.226†	-0.414**	-0.235†	-0.234†	-0.237†	-0.225†
		(0.122)	(0.189)	(0.123)	(0.123)	(0.122)	(0.123)		(0.123)	(0.127)	(0.123)	(0.123)	(0.123)	(0.122)
DMT		-0.292	-0.328	-2.384	-0.231	-0.290	-0.302		-0.244	-0.240	0.805	-0.343	-0.308	-0.225
		(1.433)	(1.453)	(1.881)	(1.442)	(1.422)	(1.451)		(1.446)	(1.455)	(1.188)	(1.473)	(1.424)	(1.460)
Ayahuasca		0.595	0.576	1.032	-0.741	0.603	0.581		0.588	0.590	1.896	0.275	0.598	0.573
		(2.109)	(2.148)	(2.122)	(1.757)	(2.131)	(2.142)		(2.106)	(2.120)	(2.285)	(2.628)	(2.144)	(2.132)
Pey/Mesc		-0.409*	-0.415*	-0.410*	-0.409*	-0.138	-0.405*		-0.414*	-0.439*	-0.408*	-0.409*	-0.677***	-0.436*
		(0.179)	(0.178)	(0.179)	(0.178)	(0.305)	(0.177)		(0.179)	(0.179)	(0.179)	(0.179)	(0.192)	(0.177)

LSD	0.0254	0.0226	0.0240	0.0244	0.0280	0.435*		0.0250	0.0195	0.0230	0.0248	0.0230	-0.197
	(0.121)	(0.120)	(0.121)	(0.121)	(0.120)	(0.191)		(0.120)	(0.121)	(0.120)	(0.121)	(0.120)	(0.137)
MDMA * Priv Hlth Ins	-0.185												
	(0.183)												
Psilocybin * Priv Hlth Ins		-0.609**											
		(0.217)											
DMT * Priv Hlth Ins			3.670										
			(2.389)										
Ayahuasca * Priv Hlth Ins				2.158									
				(3.226)									
Pey/Mesc * Priv Hlth Ins					-0.436								
					(0.390)								
LSD * Priv Hlth Ins						-0.632**							
						(0.235)							
LCPU * Pub Hlth Ins							1.074***						
							(0.207)						
MDMA * Pub Hlth Ins								0.516*					
								(0.221)					
Psilocybin * Pub Hlth Ins									0.852**				
									(0.258)				
DMT * Pub Hlth Ins										-7.354*			
										(3.220)			
Ayahuasca * Pub Hlth Ins											1.613		
											(3.026)		
Pey/Mesc * Pub Hlth Ins												0.921*	
												(0.373)	
LSD * Pub Hlth Ins													0.902***
													(0.253)

Constant	18.29^{***}	18.30^{***}	18.28^{***}	18.31^{***}	18.31^{***}	18.30^{***}	18.28^{***}	18.34^{***}	18.31^{***}	18.31^{***}	18.31^{***}	18.31^{***}	18.32^{***}	18.31^{***}
	(0.230)	(0.229)	(0.229)	(0.228)	(0.228)	(0.228)	(0.230)	(0.229)	(0.228)	(0.228)	(0.228)	(0.228)	(0.228)	(0.229)
Observations	99616	99616	99616	99616	99616	99616	99616	99616	99616	99616	99616	99616	99616	99616
R^2	0.170	0.169	0.170	0.169	0.169	0.169	0.170	0.170	0.170	0.170	0.170	0.169	0.170	0.170

Standard errors in parentheses
Source: 2008-2019 National Survey of Drug Use and Health, n=484,732
[a]Single serves as the reference category for Marital Statuses
[b]White serves as the reference category for race/ethnicity
[c]Native Indian or Alaskan Native
[d]Native Hawaiian or Pacific Islander
[e]2008 serves as the reference category for the survey year
† $p < .10$, * $p < .05$, ** $p < .01$, *** $p < .001$
